# Clinical Impact of the Line Probe Assay and Xpert^®^ MTB/RIF Assay in the Presumptive Diagnosis of Drug-Resistant Tuberculosis in Brazil: A Pragmatic Clinical Trial

**DOI:** 10.1590/0037-8682-0191-2021

**Published:** 2022-02-25

**Authors:** Afranio Kritski, Maria Martha Oliveira, Isabela Neves de Almeida, Daniela Ramalho, Monica Kramer de Noronha Andrade, Monica Carvalho, Pryscila Fernandes Campino Miranda, Margareth Pretti Dalcolmo, Jose Ueleres Braga, Tania Brígido, Eliene Mesquita, Claudia Dias, Aglae Gambirasio, Joao Baptista Souza, Anne Detjen, Patrick Peter John Phillips, Ivor Langley, Paula Fujiwara, Stephen Bertel Squire

**Affiliations:** 1 Universidade Federal do Rio de Janeiro, Faculdade de Medicina, Programa Acadêmico de Tuberculose, Rio de Janeiro, RJ, Brasil.; 2 Universidade Federal de Ouro Preto, Escola de Farmácia, Departamento de Análises Clínicas, Ouro Preto, MG, Brasil.; 3 Fundação Oswaldo Cruz, Escola Nacional de Saúde Pública, Rio de Janeiro, RJ, Brasil.; 4 Universidade do Estado do Rio de Janeiro, Rio de Janeiro, RJ, Brasil.; 5 Secretaria da Saúde do Ceará, Hospital de Messejana, Fortaleza, CE, Brasil.; 6 Secretaria de Estado de Saúde do Rio de Janeiro, Instituto Ary Parreiras, Rio de Janeiro, RJ, Brasil.; 7 Secretaria de Estado de Saúde do Rio Grande do Sul, Hospital Sanatório Partenon, Porto Alegre, RS, Brasil.; 8 Secretaria de Estado da Saúde de São Paulo, Instituto Clemente Ferreira, São Paulo, SP, Brasil.; 9 Universidade Federal do Rio de Janeiro, Programa de Engenharia Elétrica, Departamento de Engenharia Eletrônica e de Computação, Rio de Janeiro, RJ, Brasil.; 10 United Nations Children’s Fund (UNICEF), New York, USA.; 11 Institute of Clinical Trials and Methodology, MRC Clinical Trials Unit, London, United Kingdom.; 12 University of California, San Francisco, USA.; 13 Liverpool School of Tropical Medicine, Liverpool, United Kingdom.

**Keywords:** Tuberculosis, Diagnostics, Impact assessment, Molecular tests, MDR-TB

## Abstract

**Background:**

Rapid molecular methods such as the line probe assay (LPA) and Xpert^®^ MTB/RIF assay (Xpert) have been recommended by the World Health Organization for drug-resistant tuberculosis (DR-TB) diagnosis. We conducted an interventional trial in DR-TB reference centers in Brazil to evaluate the impact of the use of LPA and Xpert.

**Methods:**

Patients with DR-TB were eligible if their drug susceptibility testing results were available to the treating physician at the time of consultation. The standard reference MGIT^TM^ 960 was compared with Xpert (arm 1) and LPA (arm 2). Effectiveness was considered as the start of the appropriate TB regimen that matched drug susceptibility testing (DST) and the proportions of culture conversion and favorable treatment outcomes after 6 months.

**Results:**

A higher rate of empirical treatment was observed with MGIT alone than with the Xpert assay (97.0% vs. 45.0%) and LPA (98.2% vs. 67.5%). Patients started appropriate TB treatment more quickly than those in the MGIT group (median 15.0 vs. 40.5 days; p<0.01) in arm 1. Compared to the MGIT group, culture conversion after 6 months was higher for Xpert in arm 1 (90.9% vs. 79.3%, p=0.39) and LPA in arm 2 (80.0% vs. 83.0%, p=0.81).

**Conclusions:**

In the Xpert arm, there was a significant reduction in days to the start of appropriate anti-TB treatment and a trend towards greater culture conversion in the sixth month.

## INTRODUCTION

Tuberculosis (TB) is an infectious disease caused by a single infectious agent and is one of the top 10 fatal diseases worldwide. In 2019, the World Health Organization (WHO) reported an estimated 1.4 million deaths due to TB. Drug-resistant TB (DR-TB) continues to be a public health crisis. According to the 2019 worldwide estimate, approximately 500,000 people developed TB with rifampicin (RIF) resistance (RR) and drug resistance (DR) to the most effective first-line drug, and, of them, 78% had multidrug-resistant TB (MDR-TB)^1^. There was some global progress in testing, detection, and treatment of MDR-TB/RR-TB in 2019: 61% of people with bacteriologically confirmed TB were tested. Despite this, the importance of rapid detection of DR-TB and the adoption of correct treatment has been emphasized^1^. 

Envisaging a more efficient response to the global coinfection emergence of TB in HIV and MDR-TB cases, the WHO recommended rapid drug susceptibility testing (DST) using phenotypic methods in 2007 and molecular techniques such as the line probe assay (LPA) using the Genotype^®^ MTBDR*plus* assay (Hain Lifescience, Nehren, Germany) in 2008 and Xpert^®^ MTB/RIF assay (Cepheid^©^, Sunnyvale, USA) in 2010^2^ ^,^ ^3^.

Recently, to provide more useful data for the decision-making of TB managers, it was recommended that the incorporation of new TB diagnostic tests under routine conditions should also be adapted by the healthcare process of local healthcare systems^4^.

In 2015, the World Health Assembly endorsed the End TB Strategy proposed by the WHO, which established ambitious targets to be met by 2035. Among them, the diagnosis of TB and DR/MDR-TB was prioritized, with a focus on the systematic screening of contacts and risk groups and recommending that governments should provide universal access to DST^5^. The adoption of such strategies may help TB programs cope with the current clinical management demands, as well as the implementation of new anti-TB regimens.

Regarding the incorporation of new tests for the diagnosis of DR/MDR-TB, limited studies have analyzed the clinical impact of molecular techniques under field conditions^6^ ^-^ ^11^. Among studies that have analyzed the use of LPA, a reduction in the time interval between triage and laboratory confirmation of LPA^6^ ^,^ ^7^, the adoption of appropriate anti-TB treatment^8^ ^-^ ^10^, and a higher culture conversion rate at the sixth or eighth month of clinical follow-up^7^ ^,^ ^9^ ^,^ ^10^ were observed; however, cases of successful anti-TB treatment were described in only one study^10^.

Moreover, in a study evaluating the use of Xpert in presumed DR-TB cases^11^, a reduction in the time between triage and the adoption of appropriate treatment was reported, but no significant difference was observed in the cure rates between patients allocated to the Xpert group and those in the control group.

Despite the continuing rollout of these new tests in high-burden countries, this is the first study to conduct a pragmatic, randomized, empirical trial involving DR-TB patients to evaluate whether molecular tests would indeed improve patient-important outcomes and endpoints such as reductions in empirical treatment and community transmission^12^ ^,^ ^13^.

In Brazil, more effective TB control involves overcoming serious obstacles, including the low detection rate of DR-TB, which usually results in high morbidity and mortality, as DST is performed in only 20% of the presumed DR/MDR-TB patients^14^. Although the Bactec^TM^ MGIT^TM^ 960 system (BD Diagnostic Systems, Sparks, MD, USA) has been commercialized in Brazil, it has not been incorporated into the public healthcare system for the diagnosis of DR/MDR-TB. In 2014, Xpert was incorporated into public health and was performed for presumed TB patients, but there is no information related to the use of these new diagnostic technologies in public TB reference centers that manage DR/MDR-TB patients. Recently, in 2021, the LPA was incorporated into the Brazilian Unified Health System (SUS), different from the reality of the period in which this study was conducted^15^. 

To assist in the evaluation of the impact of incorporating molecular tests in the Brazilian Unified Health System, the International Union Against Tuberculosis and Lung Disease (The Union), through the TREAT TB initiative, the Brazilian Network of Tuberculosis Research^16^, and the Academic Tuberculosis Program of the Federal University of Rio de Janeiro, carried out a pragmatic, multicenter, prospective one-way crossover interventional trial among presumed DR-TB cases evaluated in state reference centers, called PROVE IT (registration no. RBR-4rprbd). This study aimed to compare the impact of adopting the MGIT, LPA, and Xpert tests on the diagnosis of presumed DR-TB patients relative to treatment outcomes.

## METHODS

### Study design

This study was a one-way crossover randomized trial in which each center constituted a unit of randomization and was allocated to one of two arms: arm 1, Xpert with MGIT in the first period, MGIT alone in the second period, MGIT alone in the first period, and Xpert with MGIT in the second period; and arm 2, either LPA with MGIT in the first period and MGIT alone in the second period or MGIT alone in the first period and LPA with MGIT in the second period. Owing to logistic challenges, only five sites were available, and while the randomization of the five sites proceeded as planned, this number was too small for a cluster randomized trial; therefore, the study was performed as a pragmatic, multicenter, prospective interventional trial instead. After randomization of the selected DR-TB reference centers, outpatients and hospitalized patients were tested using either the LPA, Xpert, or MGIT test.

### Setting and study period

A multicenter, pragmatic, prospective one-way crossover interventional trial was conducted at five DR-TB reference centers in four provinces in Brazil from October 2011 to May 2013. It involved inpatients and outpatients from Hospital Sanatorio Partenon, Secretaria Estadual de Saúde, Rio Grande do Sul, Hospital Messejana, Secretaria Estadual do Ceará, Instituto Estadual Ary Parreiras, and Secretaria Estadual do Rio de Janeiro, as well as outpatients from Instituto Clemente Ferreira, Secretaria Estadual de São Paulo, Centro de Referência Hélio Fraga, and Fundação Oswaldo Cruz in Rio de Janeiro.

The protocol was approved by the National Research Ethics Committee (CONEP no. 520/2011; registration no. 16571; process no. 25000.115789/2011-94) on September 29, 2011, and by the Ethics Advisory Group of the Union (no. 11/11) on May 10, 2011. The protocol was approved by the appropriate local institutional review board and ethics committee. Protocol registration was delayed due to operational issues in the Brazilian Clinical Trials Network (registration no. RBR-4rprbd).

### Participants

Eligible participants were aged 18 years or older and had experienced a cough for 3 weeks or more. Per national guidelines at the time the study was performed^17^, those with a history of DST and bacteriological confirmation of TB besides at least one of the following conditions were defined as having presumed DR/MDR-TB: (a) suspicion of retreatment, failure, or treatment default from previous anti-TB treatment and (b) HIV seropositivity or close contact with smear-positive (SSm+) MDR-TB patients, without previous anti-TB treatment. Subjects were excluded if they had (a) confirmed drug-sensitive TB, (b) refused to sign the informed consent form, or (c) harbored atypical mycobacteria.

### Test allocation by site

The randomization unit consisted of eligible DR-TB reference centers. These health units were randomly assigned to use the Xpert or MGIT assay during the first and second periods (arm 1) or to use the LPA or MGIT assay during the first and second periods (arm 2), using computer-generated allocation lists. Three sites (Hospital Sanatorio Partenon, Instituto Estadual Ary Parreiras, and Centro Referência Hélio Fraga) were allocated to arm 2, and two sites (Hospital Messejana and Instituto Clemente Ferreira) were allocated to arm 1.

In period 1, patients were enrolled from October 2011 to July 2012, and in period 2, enrollment started in August 2012 and ended in May 2013. All patients were followed up for 6 months. During the first period, LPAs and MGIT assays were performed at the Instituto Estadual Ary Parreiras and Centro Referência Hélio Fraga, and Xpert and MGIT assays were performed at the Instituto Clemente Ferreira.

In period 2, LPA and MGIT assays were performed at Hospital Parthenon, and Xpert assays with MGIT assays were performed at Hospital Messejana. When the trial was first designed, more sites were planned to improve the feasibility of the crossover cluster randomized clinical trial (RCT).

As this study was ultimately conducted with a smaller number of sites, it was not feasible to analyze the data with the unit of randomization as the health center (with only five centers); therefore, data were not analyzed as a cluster RCT but as a prospective one-way crossover interventional trial. Individuals assessing the outcomes were blinded to the interventions.

### Data collection

An impact assessment framework was used to define the key data that would be collected for this study^13^. The study did not modify any routinely administered procedures. The collected clinical data were extracted monthly from patient registers and clinical records using a study form. Patient registers had the following information about all patients included in this study: relative’s name, age, sex, address, phone number, type of patient (previous treatment classification), and date of diagnosis. Clinical and sociodemographic information was collected by healthcare workers on-site. History of tobacco smoking and alcohol use was ascertained at entry via a standardized staff-administered questionnaire (current smoker/past smoker/never smoker). The clinical samples collected in the health units were sent to a participating laboratory using local standard practice and routine; that is, samples were sent daily or twice a week. Laboratories issued results according to routine procedures. Data were collected at each site for 15 months, during each intervention phase (9 months), and at follow-up (6 months).

A standardized form was used to collect data regarding time from (1) triage (screening visit) to clinical consultation, (2) sputum collection to the release of the DST result by the laboratory, and (3) the DST results seen by the physician and adoption of appropriate TB treatment (initiation or change in the anti-TB regimen after DST results).

DR-TB and MDR-TB patients received TB treatment according to the National TB Guidelines. For the first-line regimen, RIF, isoniazid (INH), ethambutol (EMB), and pyrazinamide were administered for the first 2 months, followed by RIF and INH for 4 months. For the second-line regimen, streptomycin (SM), amikacin, EMB, levofloxacin, pyrazinamide, and terizidone were administered for the first 6 months, followed by EMB, levofloxacin, and terizidone for 12 months^17^.

### Laboratory procedures

The sites were randomized and assigned one diagnostic approach for 9 months and then switched to other diagnostic approaches. All patients were assessed using the same diagnostic approach for a particular period. According to site randomization, participants in arm 1 were assigned to have their samples analyzed by the MGIT assay alone during a 9-month period or by Xpert assays with MGIT assays in another period.

For arm 2, similar procedures were followed: patients were assigned to have their samples analyzed by MGIT assays alone or by LPA with MGIT assays. All clinical samples from the five DR-TB reference centers were sent to the local laboratories for culture, DST for first-line drugs (RIF, INH, EMB, and SM), and identification of *Mycobacterium tuberculosis complex* (Mtb complex). Tests were performed according to the local TB laboratory routine, and the techniques are fully described elsewhere^18^. The smears were stained using the Ziehl-Neelsen stain and scored according to international guidelines. Patients were classified as having SSm+ TB if any smear revealed the presence of acid-fast bacilli over 100 fields (1,000× for light microscopy and 400× for fluorescence microscopy)^18^.

As the MGIT assay was considered the reference standard, all subjects’ samples were evaluated through this method, including those allocated to the LPA or Xpert assay arms. A concentrated smear was prepared and examined, followed by culture, including mycobacterial growth indicator tubes with PANTA and OADC. Positive cultures were defined based on the detection of Mtb complexes using p-nitrobenzoic acid testing. For LPA, DNA was extracted from a portion of the decontaminated sediment, followed by multiplex polymerase chain reaction amplification and reverse hybridization using LPA, according to the manufacturer’s instructions^19^.

A four-module Xpert machine, desktop computer, and uninterrupted power supply were installed at each health unit together with a thermometer and a hygrometer^11^. No additional equipment or infrastructure was installed. Xpert assays were performed directly using sputum samples. MGIT was performed at each participant’s reference health center.

### Case definition

DR-TB patients were defined as those harboring Mtb isolates resistant to one or more drugs, and MDR-TB patients were defined as those harboring Mtb isolates resistant to RIF and INH, according to MGIT results. The MGIT results were compared with the LPA and Xpert results.

Empirical treatment was defined when, at triage, the physicians started TB treatment before receiving the DST results. Clinical and radiological improvements were assessed by the attending physicians at each site. All DR/MDR-TB patients were followed up as routinely planned according to the local algorithm. To evaluate the additional endpoints, at the second and sixth months, the local study research team, using the standardized form, checked the clinical, radiological, and laboratory data and for culture tests of sputum analysis when available.

Clinical and radiological improvement and/or culture conversion at the sixth month were considered favorable TB treatment responses; failure, death from any cause, and default were considered unfavorable results. Those who were transferred were excluded from the analysis of the TB treatment outcomes.

### Endpoints

The primary endpoint was defined as the time to initiation of an appropriate TB regimen, calculated as the time interval from triage of presumed DR-TB subjects to a TB regimen that “matched” the results of the reference standard DST.

The secondary endpoint was the proportion of presumptive TB treatment initiated at triage, culture conversion, and TB treatment outcome at the sixth month after trial enrollment.

### Sample size

For sample size calculation for the secondary endpoint, estimates of the proportion of culture conversions at the sixth month were used. Assuming a one-sided alpha of 5%, a type II error of 20% (80% power), and 40% culture negativity on MGIT assays (using data gathered from the National MDR-TB System during 2006-2007)^17^, a total of 69 patients would be required to exhibit a relative increase of 40% in LPA or Xpert (70% culture negativity on LPA or Xpert). Assuming a lack of culture results for 10% of patients at the sixth month (lost to follow-up), a total of 76 patients would be required in both periods for each comparison: LPA vs. MGIT assay and Xpert assay vs. MGIT assay. Due to the small number of clusters, the study was not adequately powered for a cluster RCT, but it had enough power when all data were analyzed together.

### Statistical analysis

Sociodemographic and clinical characteristics of the included and excluded subjects were compared. Exploratory analysis was carried out through dichotomous outcomes based on proportion calculation for groups and continuous outcomes. Means, standard deviations, and median values were calculated. The sample distribution of time periods from triage to DST results, physician decision making, initiation of appropriate TB treatment, culture conversion at the second and sixth months, and favorable outcomes were compared between the two arms: MGIT assay vs. LPA and MGIT assay vs. Xpert assay. Fisher’s exact test with mid-p correction was used for comparisons between proportions, and the Mann-Whitney U test was used to compare differences in morbidity. All statistical analyses were performed using IBM SPSS^®^ software (version 20).

## RESULTS

**FIGURE 1:**
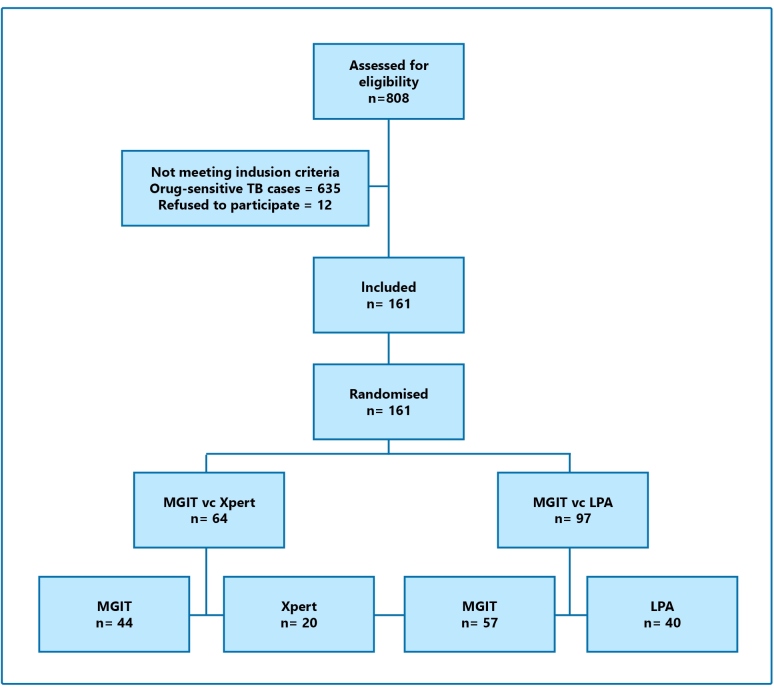
Flow diagram of presumed DR-TB with DST conducted at five reference centers, Brazil.

During the study period, 808 eligible presumed DR-TB patients with available DST results were enrolled. Among them, 647 (78.6%) were excluded: 635 with drug-sensitive TB and 12 who refused to participate (Figure 1).

Among the 161 patients included in the trial, 64 were allocated to arm 1 (MGIT, 44 vs. Xpert, 20) and 97 to arm 2 (MGIT, 57 vs. LPA, 40). Among those allocated to arm 1, in Hospital Messejana and Instituto Clemente Ferreira, 44 and 20 patients, respectively, were presumed to have DR-TB (Figure 1). Among those allocated to arm 2, there were 20, 49, and 28 presumed DR-TB patients from Instituto Ary Parreiras, Centro de Referência Hélio Fraga, and Hospital Sanatorio Partenon, respectively (Table 1).

**TABLE 1:** Distribution of DR-TB cases according to the intervention arm and study site.

	Presumed DR-TB	DR-TB cases	MGIT	Xpert	LPA
	cases with DST N	N (%)	N (%)	N (%)	N (%)
Hospital de Messejana	128	44 (34.4)	28 (63.6)	16 (80%)	
Instituto Clemente Ferreira	84	20 (23.8)	16 (36.4)	4 (20%)	
Instituto Estadual do Tórax Ary Parreiras	77	20 (25.9)	10 (17.5)		10 (25.0)
Centro de Referência Hélio Fraga	205	49 (23.9)	38 (66.7)		11 (27.5)
Hospital Sanatorio Partenon	141	28 (19.8)	9 (15.8)		19 (47.5)
**Total**	**808**	**161 (19.9)**			

**Legend:** DR-TB: drug-resistant tuberculosis; DST: drug susceptibility testing; MGIT: BactecTMMGITTM 960 system; Xpert: Xpert® MTB/RIF; LPA: line probe assay.

Among the 161 included DR-TB cases, 108 (67.1%) were male, 89 (55.3%) were aged 26-45 years, 46 (28.6%) were alcoholics, 62 (38.5%) were current tobacco smokers, and 43 (26.7%) and 24 (14.9%) had been admitted to the hospital or sent to prison in the past 2 years, respectively. HIV was diagnosed in 22 (16.5%) of 133 tested patients, and contact with DR-TB cases was reported in 59 (36.6%) cases. A total of 119 patients (73.9%) had a history of TB; among them, 29 (24.3%) had received more than three treatments. Cavities on chest radiographs were observed in 134 patients (83.3%). Smear negativity/culture positivity was identified in 24 cases (15%). These results are displayed in Table 2 **,** Table 3 **,** and Table 4, respectively.

**TABLE 2:** Sociodemographic characteristics among presumed DR-TB cases according to the intervention arm.

	Intervention Arm							
	Arm 1				Arm 2			
	MGIT	Xpert			MGIT	LPA		
Variable	N (%)	N (%)	OR* (95% CI)	P-value	N (%)	N (%)	OR* (95% CI)	P-value
**Sex**								
Male	27 (61.4)	14 (70.0)	0.68 (0.2-2.11)	p=0.50	36 (63.2)	31 (77.5)	0.49 (0.19-1.24)	p=0.13
Female	17 (38.6)	6 (30.0)	1.0		21 (36.8)	9 (22.5)	1.0	
**Age (years)**								
<25	7 (15.9)	4 (20.0)	1.0		3 (5.3)	6 (15.0)	1.0	
26-45	22 (50.0)	13 (65.0)	0.96 (0.23-3.94)	p=0.96	36 (63.2)	18 (45.0)	4.00 (0.89-17.87)	p=0.12
>45	15 (34.1)	3 (15.0)	2.85 (0.48-16.3)	p=0.27	18 (31.6)	16 (40.0)	2.25 (0.48-10.50)	p=0.50
**Skin color**								
White	22 (50.0)	5 (25.0)	0.33 (0.10-1.07)	p=0.06	45 (78.9)	23 (57.5)	2.77 (1.13-6.77)	p=0.02
Non-white	22 (50.0)	15 (75.0)	1.0		12 (21.1)	17 (42.5)	1.0	
**Smoking**								
Current	18 (40.9)	5 (25.0)	1.0		23 (40.4)	16 (40.0)	1.0	
Ex-smoker	18 (40.9)	7 (35.0)	0.71 (0.19-2.67)	p=0.61	13 (22.8)	14 (35.0)	1.54 (0.57-4.15)	p=0.39
Never	8 (18.2)	8 (40.0)	0.27 (0.06-1.12)	p=0.06	21 (36.8)	10 (25.0)	0.68 (0.25-1.83)	p=0.45
**Alcoholism (CAGE)**								
Yes	5 (11.4)	2 (10.0)	1.15 (0.20-6.52)		8 (14.0)	10 (25.0)	0.49 (0.17-1.37)	p=0.17
No	39 (88.6)	18 (80.0)	1.0	p=1.00	49 (86.0)	30 (75.0)	1.0	
**Hospitalization in the last 2 years**								
Yes	6 (13.6)	6 (30.0)	0.36 (0.10-1.33)		15 (26.3)	16 (40.0)	0.53 (0.23-1.27)	p=0.15
No	38 (86.4)	14 (70.0)	1.0	p=0.16	42 (73.7)	24 (60.0)	1.0	
**Prison in the last 2 years**								
Yes	7 (15.9)	1 (5.0)	3.59 (0.41-31.9)	p=0.41	9 (15.8)	7 (17.9)	0.85 (0.29-2.53)	p=0.78
No	37 (84.1)	19 (95.0)	1.0		48 (84.2)	32 (82.1)	1.0	
Not reported						1		
**Shelters in the last 2 years**								
Yes	0 (0.0)	0 (0.0)	-	-	5 (8.9)	2 (5.0)	1.86 (0.34-10.1)	p=0.69
No	43 (100.0)	20 (100.0)			51 (91.1)	38 (95.0)	1.0	
Not reported	1	-			1			

**Legend:** OR: odds ratio; CI: confidence interval; MGIT: BactecTMMGITTM 960 system; Xpert: Xpert® MTB/RIF; LPA: line probe assay.

**TABLE 3:** Clinical, laboratory, and radiological characteristics among presumed DR-TB at triage according to the intervention arm.

	Intervention Arm							
	Arm 1				Arm 2			
	MGIT	Xpert			MGIT	LPA		
Variable	N (%)	N (%)	OR* (95% CI)	P-value	N (%)	N (%)	OR* (95% CI)	P-value
**Contact with Pulmonary TB**								
Yes	18 (52.9)	7 (50.0)	1.12 (0.32-3.90)		18 (40.0)	16 (59.3)	0.45 (0.17-1.21)	p=0.11
No	16 (47.1)	7 (50.0)	1.0	p=1.00	27 (60.0)	11 (40.7)	1.0	
Not reported	10	6	-		12	13		
**Comorbidities**								
Yes	24 (57.1)	11 (57.9)	0.97 (0.32-2.90)	p=0.95	27 (47.4)	29 (72.5)	0.34 (0.14-0.81)	p=0.01
No	18 (42.9)	8 (42.1)	1.0		30 (52.6)	11 (27.5)	1.0	
Not reported	2	1						
**TB in the past**								
Yes	32 (74.4)	15 (83.3)	0.58 (0.14-2.39)	p=0.52	44 (78.6)	28 (70.0)	1.57 (0.62-3.98)	p=0.33
No	11 (25.6)	3 (16.7)	1.0		12 (21.4)	12 (30.0)	1.0	
Not reported	1	2	-		1			
**Number of previousTB treatments**								
≥3	11 (34.4)	4 (26.7)	1-44 (0.37-5.59)	p=0.74	7 (15.9)	7 (25.0)	0.56 (0.17-1.84)	p=0.34
<3	21 (65.6)	11 (73.3)	1-0		37 (84.1)	21 (75.0)	1.0	
Not applicable	12	5						
**Treatment outcome in the last TB episode**								
Cure	8 (25.8)	2 (13.3)	0.44 (0.08-2.40)	p=0.57	15 (33.3)	9 (32.1)	0.94 (0.34-2.59)	p=0.91
Failure	23 (74.2)	13 (86.7)	1.0		30 (66.7)	19 (67.9)	1.0	
Not applicable	1	5						
Not reported	12	0						
**Weight loss**								
Yes	21 (47.7)	15 (75.0)	4.10 (1.17-14.36)	p=0.02	42 (73.7)	35 (87.5)	2.33 (0.76-7.11)	p=0.12
No	23 (52.3)	4 (25.0)	1.0		14 (24.6)	5 (12.5)	1.0	
Not reported		1			1	-		
**Expectorated cough**								
Yes	40 (90.9)	18 (90.0)	1.11 (0.18-6.63)		48 (84.2)	34 (85.0)	0.94 (0.30-2.89)	p=0.91
No	4 (9.1)	2 (20.0)	1.0	p=1.00	9 (15.8)	6 (15.0)	1.0	
Not reported			-		0	0		
**Hemoptysis**								
Yes	10 (22.7)	6 (30.0)	0.68 (0.20-2.25)	p=0.54	19 (33.3)	14 (35.0)	0.92 (0.39-2.17)	p=0.86
No	34 (77.3)	14 (70.0)	1.0		38 (66.7)	26 (65.0)	1.0	
Not reported			-					
**Sweating**								
Yes	23 (54.7)	14 (70.0)	1.92 (0.62-5.98)	p=0.25	39 (68.4)	29 (72.5)	1.0	
No	19 (45.3)	6 (30.0)	1.0		18 (31.6)	9 (22.5)	1.48 (0.58-3.78)	p=0.40
Not reported	2	0			-	2 (5.0)	-	p=0.38
**Fever**								
Yes	26 (60.4)	15 (75.0)	1.92 (0.62-5.98)	p=0.26	38 (66.7)	27 (67.5)	1.0	
No	17 (39.6)	5 (25.0)	1.0		18 (31.6)	12 (30.0)	1.06 (0.44-2.85)	p=0.88
Not reported	1				1 (1.8)	1 (2.5)	0.71 (0.04-11.8)	p=1.00
**Appetite loss**								
Yes	25 (58.1)	15 (75.0)	2.16 (0.66-7.02)	p=0.19	42 (73.7)	27 (67.5)	1.34 (0.55-3.27)	p=0.50
No	18 (41.9)	5 (25.0)	1.0		15 (26.3)	13 (32.5)	1.0	
Not reported	1		-					
**Dyspnea**								
Yes	14 (42.4)	3 (15.0)	0.36 (0.09-1.45)	p=0.14	44 (77.2)	21 (52.5)	0.34 (0.14-0.83)	p=0.01
No	29 (57.6)	17 (85.0)	1.0		13 (22.8)	18 (45.0)	1.0	p=0.66
Not reported	1	-			-	1 (2.5)	-	
**Anti-HIV testing performed?**								
Yes	34 (82.9)	16 (84.2)	0.91 (0.20-3.99)	p=1.00	53 (94.6)	30 (75.0)	5.8 (1.5-23.07)	p=0.006
No	7 (17.1)	3 (15.8)	1.0		3 (5.4)	10 (25.0)	1.0	
Not reported	3	1			1			
**HIV testing result**								
Positive	3 (9.1)	3 (18.8)	0.43 (0.07-2.43)	p=0.37	10 (18.9)	6 (20.7)	0.89 (0.28-2.76)	p=0.84
Negative	30 (90.0)	13 (81.3)	1.0		43 (81.1)	23 (79.3)	1.0	
Not reported	11	4	-		4	1		
**Smearmicroscopy**								
Positive	37 (84.1)	18 (90.0)	0.58 (0.11-3.11)	p=0.70	47 (83.9)	35 (87.5)	0.74 (0.23-2.42)	p=0.62
Negative	7 (15.9)	2 (20.0)	1.0		9 (16.1)	5 (12.5)	1.0	
Not reported					1			
**Cavity**								
Yes	33 (75.0)	19 (95.0)	0.15 (0.01-1.32)		46 (80.7)	36 (90.0)	0.46 (0.13-1.58)	p=0.21
No	11 (25.0)	1 (5.0)	1.0	p=0.08	11 (19.3)	4 (10.0)	1.0	
**Chest X-ray images**								
Typical	40 (90.9)	19 (95.0)	0.52 (0.05-5.03)	p=1.00	49 (86.0)	38 (95.0)	2.38 (0.45-12.49)	p=0.50
Compatible	4 (9.1)	1 (5.0)	1.0		6 (10.5)	2 (5.0)	1.0	
Atypical	0	0	-		2 (3.5)	0	-	p=0.63

**Legend:** OR: odds ratio; CI: confidence interval; MGIT: BactecTMMGITTM 960 system; Xpert: Xpert®MTB/RIF; LPA: line probe assay.

**TABLE 4:** Distribution of time from triage to clinical outcomes according to study arm.

	Arm 1			Arm 2		
Time (days) from triage to	MGIT	Xpert	P-value	MGIT	LPA	P-value
	N (IQR)	N (IQR)		N (IQR)	N (IQR)	
Sputum collection	0 (0-0)	0 (0-0.75)	0.71	0 (0-0)	0 (0-1.00)	0.87
DST results release	32.5 (27.2-47.0)	2.2 (0-2.5)	<0.001	34.0 (22-62.0)	9.0 (7-15.0)	<0.001
DST results received by the physician	55.5 (38.2-103.5)	7.0 (3-13.3)	<0.001	40.2 (27.2-65.7)	30.0 (9-33.2)	<0.001
Changing treatment	47 (35-87.7)	30.0 (6.7-42.7)	0.005	69.0 (46-84.0)	61.0 (34-121.0)	0.99
Treatment that matches with standard reference DST	40.5 (28.7-76.7)	15.0 (4.5-38.5)	0.01	54.0 (17-80.5)	61.4 (19.7-104.7)	0.78

**Legend:** IQR: interquartile range; DST: drug susceptibility testing; MGIT: BactecTMMGITTM 960 system; Xpert: Xpert® MTB/RIF; LPA: line probe assay.

In comparison to MGIT, among the 20 patients evaluated by Xpert, resistance to RIF was identified in 15 samples and confirmed in 13 (11 MDR-TB and 2 RR), and five samples were false sensitive to RIF.

Additionally, in comparison to MGIT, among 40 patients evaluated by LPA, resistance to RIF was identified in 27 samples and confirmed in 23; sensitivity to RIF was identified in 8 and confirmed in 1. Resistance to INH was identified in 37 samples and confirmed in 35 patients (34 MDR).

In summary, false-positive results for RIF resistance were observed in 13% (95% confidence interval [CI]: 6.3-45.9) of patients in the Xpert arm and 14.8% (95% CI: 5.3-33.1) of patients in the LPA arm; when comparing sociodemographic, clinical, laboratory, and radiological characteristics, the occurrence of weight loss was significantly more frequent in the Xpert arm than in the MGIT arm (75.0% vs. 47.7%). White race (57.5% vs. 78.9%), HIV testing (75.0% vs. 94.6%), and dyspnea (52.5% vs. 77.2%) were less frequent, but comorbidity was higher (72.5% vs. 47.4%) in the LPA arm than in the MGIT arm (Table 2 and Table 3).

Regarding the primary endpoint, the median time (in days) was lower in the Xpert arm than in the MGIT arm from triage to the adoption of appropriate TB treatment (15.0 vs. 40.5). Additionally, the median interval time (in days) was also lower in the Xpert arm from triage to the release of test results by the laboratory (2.2 vs. 32.5) and test results seen by physicians (7.0 vs. 55.5) (Table 4).

For arm 2, the median time interval (in days) was lower for the LPA arm than for the MGIT arm from triage to test results being released by the laboratory (9.0 vs. 34.0) and test results seen by physicians (30.4 vs. 40.2) but not the adoption of an appropriate TB treatment (Table 4).

Comparing the approaches to clinical decision making, a higher proportion of presumed DR-TB patients allocated to the LPA arm (35% vs. 12%) received the second-line regimen at the health unit of origin than those in the MGIT arm. At triage, among the subjects evaluated in the two arms, a higher rate of empirical treatment was observed with MGIT alone than with Xpert assays (97.0% vs. 45.0%) and LPA (98.2% vs. 67.5%) (Table 5).

**TABLE 5:** TB treatment adopted at triage and during follow-up among DR-TB patients according to the intervention arm.

	Intervention Arm							
Variable	Arm 1				Arm 2			
	MGIT	Xpert	OR* (95%CI)	P-value	MGIT	LPA	OR* (95%CI)	P-value
	N (%)	N (%)			N (%)	N (%)		
**Empirical** treatment								
Yes	43 (97.7)	9 (45.0)			56 (98.2)	27 (67.5)	26.9 (3.35-216.9)	
1st line drug	31	17	52.5 (6.0-460.08)	p<0.01	50	26		p<0.001
2nd line drug	13	3	1.0		7	14	1.0	
No	1 (2.3)	11 (55.0)			1 (1.8)	13 (32.5)		
**TB treatment adopted at triage**								
Maintained the regimen prescribed earlier by health unit	17 (38.6)	8 (40.0)	0.94 (0.32-2.78)	p=1.00	29 (50.9)	14 (35.0)	1.92 (0.83-4.41)	
Started new regimen	27 (61.4)	12 (60.0)	1.0		28 (49.1)	26 (65.0)	1.0	p=0.12
TB treatment that matches withreference standard DST(including those withempirical treatment)								
Yes	35 (100)	14 (82.4)	-	p=0.04	51 (100.0)	29 (93.5)	-	p=0.14
No	0 (0.0)	3 (17.6)			0 (0.0)	2 (6.5)		
**SSM 2nd month**								
Positive	9 (27.3)	3 (20.0)	1.5 (0.34-6.58)	p=0.72	16 (43.2)	11 (35.5)	1.38 (0.52-3.7)	p=0.51
Negative	24 (72.7)	12 (80.0)	1,0		21 (56.8)	20 (64.5)	1.0	
Not reported	11	5			20	9		
**SSM 6th month**								
Positive	6 (19.4)	0 (0)		p=0.16	4 (14.8)	10 (34.5)	0.33 (0.08-1.22)	p=0.09
Negative	25 (80.6)	12 (100)	-		23 (85.2)	19 (65.5)	1.0	
Not reported	13	8			30	11		
**Culture 2nd month**								
Positive	22 (64.7)	5 (35.7)	3.3 (0.89-12.1)	p=0.06	20 (54.1)	13 (50.0)	1.17 (0.43-3.21)	p=0.75
Negative	12 (35.3)	9 (64.3)	1.0		17 (45.9)	13 (50.0)	1.0	
Not reported	10	6			3	31		
**Culture 6th month**								
Positive	6 (29.7)	1 (9.1)	2.60 (0.27-24.6)	p=0.39	6 (17.0)	6 (20.0)	0.86 (0.24-3.01)	p=0.81
Negative	23 (79.3)	10 (90.9)	1.0		29 (83.0)	25 (80.0)	1.0	
Not reported	15	9	-		22	9		
**Any change in TB treatment after the triage**								
Yes	34 (77.3)	14 (70.0)	1.45 (0.44-4.78)	p=0.53	43 (75.4)	25 (65.8)	1.59 (0.64-3.93)	p=0.30
No	10 (22.7)	6 (30.0)	1.0		14 (24.6)	13 (34.2)	1.0	
Not reported	-	-			-	2		
**Adverse reaction**								
Yes	10 (23.8)	4 (22.2)	1.09 (0.29-4.09)	p=1.00	5 (11.4)	7 (18.9)	0.54 (0.15-1.90)	p=0.36
No	32 (76.2)	14 (77.8)	1.0		39 (88.6)	30 (81.1)	1.0	
Not applicable	2	2			13	3		
**Treatment outcome at6th month**								
Favorable	35 (79.5)	17 (85.0)	0.68 (0.16-2.86)		43 (76.8)	29 (76.3)	1.02 (0.38-2.7)	p=0.95
Unfavorable	9 (20.5)	3 (15.0)	1.0	p=0.74	13 (23.2)	9 (23.7)	1.0	
Lost of follow-up	3	1			8	1		
Failure	6	1			3	7		
Death	0	1			2	1		
Transferred					1	2		

**Legend:** SSM: sputum smear microscopy; OR: odds ratio; MGIT: BactecTMMGITTM 960 system; Xpert: Xpert®MTB/RIF; LPA: line probe assay.

At the sixth month, 26 (16.1%) had an adverse event, 17 (10.6%) failed treatment, 13 (8.0%) were lost to follow-up, 3 (1.8%) died, and 3 (1.8%) had been transferred to another healthcare unit. Among the 158 subjects who had been followed up, a favorable response was seen in 124 (78.5%), and culture conversion by the sixth month after trial enrollment occurred in 81.4% (79/97) of patients.

During anti-TB treatment, for arm 1, the following variables did not differ significantly among subjects allocated to the Xpert and MGIT arms: culture conversion at the sixth month (90.9% vs. 79.3%, p=0.39), occurrence of an adverse event (22.2% vs. 23.8%), and favorable treatment outcome (85.0% vs. 79.5%). Similar results were observed for arm 2 among subjects allocated to the LPA and MGIT arms: culture conversion by the sixth month (80.0% vs. 83.0%, p=0.81), occurrence of an adverse event (18.9% vs. 11.4%), and favorable treatment outcome (76.3% vs. 76.8%) (Table 5).

## DISCUSSION

We carried out a pragmatic, multicenter, prospective interventional trial to evaluate the clinical impact of the adoption of the Xpert assay and LPA on the diagnostic and treatment cascades of DR/MDR-TB patients attending DR-TB reference centers in a high-burden country, in conditions similar to those observed within routine health services and in patients who were similar to those who would need treatment in the future. The aim was to answer questions regarding the applicability of new technologies in the healthcare system, extending beyond the issues related to efficacy typically evaluated in explanatory clinical trials. The time (days) from triage to DST test result release and results received by clinicians were lower in the Xpert and LPA arms than in the MGIT arm, similar to the results reported by other studies^6^ ^-^ ^10^ involving LPA and by Padayatchi et al. (2016) using the Xpert assay.

However, the time (days) from triage to the adoption of a TB treatment that matched the standard reference DST result was shorter in the Xpert arm, consistent with Padayatchi et al. (2016), but not in the LPA arm, contrary to the results reported in other studies^8^ ^-^ ^10^ ^,^ ^20^, wherein a shorter time from triage to the start of TB treatment in patients was observed in the LPA group.

A higher percentage of negative cultures in the sixth month after enrollment was observed in the Xpert arm but not in the LPA arm. These results differed from those reported elsewhere^7^ ^,^ ^9^ ^,^ ^10^, where a lower proportion of negative cultures in the sixth or eighth month was observed after screening with LPA.

The greater conversion of culture in the sixth month in the Xpert arm may result from the quicker adoption of an appropriate treatment regimen observed in the Xpert arm than in the MGIT arm. This scenario did not occur when comparing the LPA and MGIT assays. The transport of the samples to distant places and the delivery of results were carried out by a motorcycle courier. The Xpert arm did not require a laboratory and specialized team; therefore, the test was performed locally. This factor influenced the speed of DST results seen by physicians. Recently, Albert et al. (2016) noted that the computerization of the diagnostic investigation process of TB plays a central role in the incorporation of new diagnostic technologies, facilitating the transfer of test results to the clinical area and circumventing the difficulties identified in our study^4^.

For the MGIT groups, a greater proportion of patients were treated empirically (98%) than those in the Xpert (45%) and LPA (67%) groups. This finding could be explained by the delay in the release of phenotypic drug sensitivity results. Thus, clinicians needed to initiate treatment solely based on smear microscopy results, history of previous anti-TB regimens used, clinical symptoms, and chest X-ray findings to treat patients more quickly, as delayed treatment would aggravate the disease and increase the risk of death, in addition to maintaining the chain of transmission^21^.

In this study, at the sixth-month post-trial enrollment, the proportion of favorable outcomes with anti-TB treatment was similar in the Xpert and LPA arms, as compared to that in the MGIT arm; this differs from the results reported by Singla et al. and Eliseev et al., in which a greater proportion of favorable outcomes was observed with LPA, but is consistent with Padayatchi et al., who did not report this impact with the Xpert assay^10^ ^,^ ^11^ ^,^ ^20^. These discordant results may stem from the fact that both the Xpert and LPA arms, compared to the MGIT arm, had similar rates of correct treatment based on the results of the standard reference DST, despite a higher proportion of empirical treatments in the MGIT arm. Additionally, due to the small sample size, there was an insufficient number of patients to show a difference.

In the Xpert arm, 73% of patients who harbored Mtb complex isolates resistant to RIF had MDR-TB, while in the LPA arm, 85.2% were classified as having MDR-TB. Therefore, LPA was more likely to correctly classify MDR-TB according to the reference standard DST (MGIT) results. These results suggest that LPA may be a useful technology for the diagnosis of DR-TB. However, it is necessary to increase the efficiency of the transfer of laboratory results to the clinical team, computerize the processes, and increase the ability to promptly contact patients to initiate TB treatment.

Conversely, there is a great advantage in using Xpert compared with MGIT assays and LPA. The latter techniques can only be performed in central laboratories, requiring specialized laboratory technicians, whereas Xpert assays can be performed in less complex laboratories; therefore, these laboratories are situated closer to patient care centers.

In this study, false-positive results for RIF resistance were similar in the Xpert and LPA arms (13% and 14.8%, respectively). These results are higher than those reported in the literature (5%-12%). As the level of resistance (according to the MIC)^22^ was not measured in the reference standard DST, and the presence of mixed infection or heteroresistance^23^ in the respiratory samples of patients with DR-TB was not analyzed, we cannot rule out the variables that would clarify the high proportion of false positives attained in this case.

Although the proportion of HIV-infected patients observed in the LPA arm (18%) was higher than that in the Xpert arm (8%), there was no significant difference between the smear-negative TB and MDR-TB cases in the Xpert and LPA arms. The literature reports^12^ ^,^ ^13^ ^,^ ^24^ ^,^ ^25^ that HIV-infected patients with smear-negative TB are commonly treated empirically, generating unnecessary costs and more toxicities due to inadequate treatment. Although this finding was not observed in this series, the proportion of unfavorable outcomes in HIV-infected patients with MDR-TB was usually high. The mortality rate in these patients may reach 25% in populations with a high prevalence of HIV infection, which is related to the delay in diagnosis^24^ ^-^ ^28^.

In this pragmatic trial, as the unit of randomization consisted of health units, with a small number of presumed DR-TB patients included in the triage in each participant’s health unit, differences in patient characteristics were observed between the Xpert, LPA, and MGIT arms. In the Xpert arm, a greater rate of weight loss was observed. In the LPA arm, there was a higher proportion of patients with comorbidities, and in the MGIT evaluation, more patients were white, had dyspnea, and underwent HIV testing. These results represent a limitation of this study. The higher frequency of dyspnea occurrence among patients allocated to the MGIT arm than those allocated to the LPA arm may be a consequence of a poor interpretation of fatigue by the patient, as a specific data collection instrument to measure dyspnea was not used. Although a higher occurrence of comorbidities in the LPA arm was observed, no significant association of this factor with unfavorable outcomes (adverse effects, death) was observed, likely due to the small number of patients included.

Another important finding is that the greater conversion of culture in the sixth month in the Xpert arm may have resulted from the quicker adoption of an appropriate treatment regimen observed in the Xpert arm than in the MGIT arm. This scenario did not occur when comparing the LPA vs. MGIT assay; as LPA was performed in culture rather than in clinical samples, and the transport of the samples to distant places and the delivery of results were carried out by a motorcycle courier. The Xpert arm did not require a laboratory or specialized team, used clinical samples, and the test was performed locally.

The strengths of this study include the following: (a) this was a pragmatic RCT that enrolled presumed DR/MDR-TB subjects attending five DR-TB reference sites in four provinces under routine conditions; (b) the personnel performing the Xpert, LPA, or MGIT assays were unaware of the patients’ clinical or radiographic findings; and (c) only patients with culture-confirmed TB who underwent DST were included.

A limitation of the study is that it relied on a small sample size for patients with DR-TB. This limits the statistical analysis and power of the study, and the results may not be useful for generalizing the entire country. In addition, there might have been a confounding effect of time since MGIT assays were always conducted in the second period. Discordant results between MGIT assays and Xpert assays or LPA were not evaluated by sequencing. No follow-up information was collected from DR-TB suspects referred to the reference center without bacteriological confirmation.

In conclusion, the incorporation of rapid molecular tests reduced the proportion of empirical treatment and accelerated correct therapeutic decision-making and treatment initiation. Improvement of the flow of DR/MDR-TB diagnosis is warranted to optimize the use of new technologies. At 6 months post-enrollment, the rate of culture conversion was higher in the Xpert arm; however, there was no difference in treatment outcomes between patients allocated to the Xpert and LPA arms and those in the MGIT arm.
